# Exploitable Lipids and Fatty Acids in the Invasive Oyster *Crassostrea gigas* on the French Atlantic Coast

**DOI:** 10.3390/md14060104

**Published:** 2016-05-24

**Authors:** Flore Dagorn, Aurélie Couzinet-Mossion, Melha Kendel, Peter G. Beninger, Vony Rabesaotra, Gilles Barnathan, Gaëtane Wielgosz-Collin

**Affiliations:** 1 Faculté des Sciences Pharmaceutiques et Biologiques, Université de Nantes, Groupe Mer, Molécules, Santé-EA 2160, Institut Universitaire Mer et Littoral FR3473 CNRS, 9 rue Bias, BP 53508, F-44035 Nantes Cedex 1, France; flore.dagorn@gmail.com (F.D.); aurelie.couzinet-mossion@univ-nantes.fr (A.C.-M.); melha.kendel@gmail.com (M.K.); vony.rabesaotra@univ-nantes.fr (V.R.); Wielgosz-Collin@univ-nantes.fr (G.W.-C.); 2 Faculté des Sciences et des Techniques, Université de Nantes, Groupe Mer, Molécules, Santé-EA 2160, Institut Universitaire Mer et Littoral FR34473 CNRS, 2 rue de La Houssinière BP 92208, F-44322 Nantes Cedex 3, France; Peter.Beninger@univ-nantes.fr

**Keywords:** *Crassostrea gigas*, fatty acids, health and nutrition, bivalve, mollusc, non-methylene interrupted fatty acids, plasmalogens, phospholipids, seasonal variations

## Abstract

Economic exploitation is one means to offset the cost of controlling invasive species, such as the introduced Pacific oyster (*Crassostrea gigas* Thunberg) on the French Atlantic coast. Total lipid and phospholipid (PL) fatty acids (FAs) and sterols were examined in an invasive population of *C. gigas* in Bourgneuf Bay, France, over four successive seasons, with a view to identify possible sources of exploitable substances. The total lipid level (% dry weight) varied from 7.1% (winter) to 8.6% (spring). Of this, PLs accounted for 28.1% (spring) to 50.4% (winter). Phosphatidylcholine was the dominant PL throughout the year (up to 74% of total PLs in winter). Plasmalogens were identified throughout the year as a series of eleven dimethylacetals (DMAs) with chain lengths between C_16_ and C_20_ (up to 14.5% of PL FAs + DMAs in winter). Thirty-seven FAs were identified in the PL FAs. Eicosapentaenoic acid (20:5*n*-3 EPA/7.53% to 14.5%) and docosahexaenoic acid (22:6*n*-3 DHA/5.51% to 9.5%) were the dominant polyunsaturated FAs in all seasons. Two non-methylene-interrupted dienoic (NMID) FAs were identified in all seasons: 7,13-docosadienoic and 7,15-docosadienoic acids, the latter being present at relatively high levels (up to 9.6% in winter). Twenty free sterols were identified, including cholesterol at 29.9% of the sterol mixture and about 33% of phytosterols. *C. gigas* tissues thus contained exploitable lipids for health benefits or as a potential source of high-quality commercial lecithin.

## 1. Introduction

The Pacific oyster, *Crassostrea gigas*, was introduced for cultivation to the French Atlantic coasts in the 1970s to replace the Portuguese oyster *Crassostrea angulata*, which was decimated by two epidemics in the 1960s [1]. With the subsequent increase in near-shore seawater temperatures, cultured populations began to extensively colonize the French Atlantic coast, even fouling the oyster-farming sites themselves [2,3,4,5,6], with biomasses exceeding 50 kg·m^−2^ [6]. Among the most affected sites, invasive *C. gigas* is reported to constitute more than 70% of the total oyster biomass of the commercial oyster-producing Bourgneuf Bay [3,7,8]. Since the invasive and the cultured *C. gigas* are sympatric, it is certain that trophic competition also takes place, negatively affecting the oyster-culture potential. Profitable exploitation is a possible cost-effective avenue of remediation here and in the other *C. gigas*-invaded habitats.

In a previous study, we showed the potential economic value of another invasive species, *Crepidula fornicata*, which contained high added-value lipids such as a marine lecithin rich in phosphatidylcholine, and *n*-3 polyunsaturated fatty acids (PUFAs) [9]. Long-chain *n*-3 PUFAs, mainly eicosapentaenoic acid (EPA) and docosahexaenoic acid (DHA), are dietary lipids with an array of health benefits throughout life [10,11]. Phospholipid (PL)-bound *n*-3 PUFAs are of high nutritional value because of they are as bioavailable as their counterparts in conventional fish oils where *n*-3 PUFAs are bound to triacylglycerols [12]. PUFA supply from aquaculture sources has also increased over the past decades, making up for the shortfalls from fishery supplies [13,14]. Although EPA and DHA are known to be beneficial in the prevention or treatment of several diseases [15], levels of DHA and EPA are often low in Western diets [16]. This has led to the commercialization of EPA-DHA supplements, which thus consume even more of the dwindling fish oil resource [14]. Although the lipid and fatty acid contents of *C. gigas* have been investigated previously, these studies were either confined to specific body parts [17], or preliminary analyses showed that we could achieve comparatively finer resolution [18,19,20]. We therefore present the results of a detailed study of the wild *C. gigas* lipids, with a view to the future simultaneous exploitation and remediation of the invasive populations.

## 2. Results and Discussion

### 2.1. Seasonal Lipid Content and Lipid Class Composition

Total tissue lipid contents (% dry weight, DW) and percentages of the main lipid classes of *C. gigas* over the four seasons are reported in Table 1.

Lipid content varies only slightly throughout the year, from 7.1% (winter) to 8.6% DW (spring). Similar seasonal variations (from 7.8% to 8.7% DW) were observed for *C. gigas* introduced and farmed in Irish waters [21]. However, seasonal variations may be more pronounced if the species is cultured off-shore (8% to 14% DW) [20].

In the present study, non-polar lipids (triacylglycerols, sterols and free fatty acids) (FAs) accounted for 40% (winter) to 64.5% (spring) of total lipids, while PL levels occurred at 50.4% in winter and 28.1% in spring. Glycolipids ranged from 7.4% (spring) to 12.4% (summer). These high proportions of neutral lipids (NLs) and PLs are in agreement with the literature [20,22,23].

### 2.2. Seasonal Phospholipid Class Composition

Throughout the year, phosphatidylcholine (PC) was the main PL class, at 39.7% to 74% of total PLs, the highest level being observed in winter (Table 2). The lowest level observed in spring is in accordance with values reported for *C. gigas* harvested in France at the same season [23,24]. To our knowledge, a seasonal variation of PL class composition has never been reported for *C. gigas*.

The commercially active component of lecithin is PC. However, commercial ‘lecithin’ is often simply a mixture of PLs with a relatively high PC content, of unspecified FA chain length. In contrast, the *Crassostrea gigas* PLs contain a high percentage of PC, as well as long-chain PUFAs, such that the PLs may be considered a potential source of high-quality lecithin.

Lysophosphatidylcholine was present at 2.9% to 5.4%, suggesting the occurrence of phospholipases in all seasons. Phosphatidylserine ranged from 1.6% (winter) to 5.7% (autumn). Phosphatidylinositol and phosphatidylglycerol were not observed in the samples; this was verified using two-dimensional thin-layer chromatography against known standards. Interestingly, cardiolipin (CL) occurred in all seasons at significant levels (4.9%–10.7%). In mammalian tissues, CL is found almost exclusively in the inner mitochondrial membranes, mediating various respiratory functions [25,26]. The occurrence and possible functions of CL has been studied in detail in some marine bivalves, including *Pecten maximus*, *Mytilus edulis* and *C. gigas,* which contain a unique CL with four docosahexaenoyl chains; it is suggested that this is a specific adaptation in bivalves in response to variations in environmental conditions [27].

The unusually high levels of ceramide aminoethylphosphonate (CAEP) in all seasons except winter (up to 24.6% of PLs) are notable. This phosphonosphingolipid is widely distributed among molluscs [28], including in *C.* *gigas* tissues and especially hemocytes [29,30]. Throughout the year, the levels of phosphatidylethanolamine (PE) were surprisingly low (approx. 4%–18%) in *Crassostrea gigas*, compared to previously published data for other marine bivalves (32%–41%, [31]). However, our results report significant levels of CL, as well as twice as much CAEP, suggesting that the disparity may be methodological.

### 2.3. Seasonal Phospholipid Fatty Acid Composition

Fatty acid (FA) composition, determined as fatty acid methyl esters (FAMEs), is given in Table 3. In addition to the FAMEs, the resulting mixture also contained eleven fatty aldehyde dimethylacetals (DMAs), which were readily identified from their mass spectra showing a characteristic intense fragment ion at *m*/*z* 75 ([(CH_3_O)_2_ − CH]^+^) and an ion corresponding to [M − 31]^+^ [9,31]. Thirty-eight FAs with chain lengths between C_14_ and C_24_ were identified in the PLs.

The PL FA mixture consisted of SFAs (29.6% to 40.7% of the total FA + DMA mixture), MUFAs (19.5%–23.4%), PUFAs (34.5%–38.7%), and DMAs (3.8%–14.5%). SFAs included the dominant palmitic acid (14.6%–23.8%) and several branched FAs, including *iso* and *anteiso* FAs. The 11-18:1, 11-20:1 and 13-20:1 acids were the most abundant among the monounsaturated FAs. The level of 11-18:1 acid was relatively steady at 4.5% to 5.7%, except in spring where it dropped to 2.5%. Similarly, the level of 13-20:1 remained between 5.4% and 5.8%, except in spring where it decreased to 2.2%. On the contrary, the 11-20:1 acid had its highest level in spring (7.2%). This corresponds to the period in which *C. gigas* gametogenesis is initiated at this site [32].

The conventionally dominant PUFAs, eicosapentaenoic 20:5*n*-3 (EPA), and 22:6*n*-3 (DHA) acids, were present in all seasons at high levels, from 7.5% (summer) to 14.5% (spring) and 5.5% (spring) to 9.5% (summer), respectively. The percentage of *n*-3 PUFAs was markedly higher than that of *n*-6 PUFAs. According to literature (Table 4), EPA and DHA levels in oyster lipids (TLs or PLs) varied greatly depending on harvesting country, temperature and season. EPA levels varied from 7.6% to 22.4% in PLs described in literature and from 7.5% to 15.4% in the present study, which is in the same order of magnitude. DHA highest percentages (35.8% TLs and 32.5% PLs) were analyzed in oysters collected in warm water during winter [33,34] but lower levels, more in agreement with the present study, were described in oysters from Europe. The data presented here thus agree with those of the literature, and confirm that these *n*-3 PUFA levels appear typical of *C. gigas*. The abundant invasive oyster biomass could thus be a useful source of *n*-3 PUFAs, which appear to have positive clinical effects [15].

Surprisingly, the third most abundant FA in PLs was a non-methylene-interrupted (NMI) diunsaturated FA, 22:2*n*-7,15, occurring at 9.6% (winter) and 9.0% (spring). Two other NMI FAs were present, 22:2*n*-9,15 (1%–2%) and the rare 21:2*n*-8,14 as traces. Such diunsaturated NMI FAs occur commonly in marine invertebrates [35], especially in various molluscs, including oysters [20,23,24,29,36] but generally at lower levels (Table 4).

They are known to occur mainly in PLs, especially in plasmalogens (1-alkenyl-2-acyl ether glycerophospholipids) [36], shown here by eleven C_16_ and C_20_ fatty aldehyde DMAs in the lipids of *C. gigas,* at 0.6% to 1.9%. The unusual unsaturation pattern of NMI FAs provides cell membranes with increased resistance to oxidative processes and microbial lipases, relative to the common PUFAs [35,36,38,39]. The sharp decrease in NMI+DMA levels observed in summer for *C. gigas* of the present study, contrasts with the stable levels observed throughout the year in the warm-water *C. corteziensis* [34].

### 2.4. Free Sterol Composition

The free sterol fraction was the main component of the neutral lipids, especially in winter. The seasonal total free sterol composition is presented in Table 5.

Cholesterol was the most abundant sterol at relatively low levels from 21.4% (summer) to 35.8% of the unsaponifiable fraction (spring). The notable decrease in the summer value probably reflects the result of gamete emission, as was also observed for the autumn values for this species in the more southern Venice lagoon [19].

Other major sterols present were brassicasterol (9.2%–12.8%), 24-methylenecholesterol (ostreasterol, 8.4%–14%), β-sitosterol (6.0%–10.1%). Previous studies on seasonal variation of sterol composition of *C. gigas* (whole body or hematocyte membranes) have reported similar results [19,29] as well as studies on *C. corteziensis* and *C. virginica* [34,40]. In addition, 22*E*-dehydrocholesterol (5.6%–8.4%), campesterol (5.5%–6.9%) have also been previously described in oysters, but not cholestanol (3.9%–6.7%) [34,40].

Although 4-methylsterols are well known to be typical dinoflagellate sterols, they may also be found in diatoms, and are indicative of a trophic link [41,42,43]. Interestingly, 4-methylsterols and β-sitosterol have hypolipidemic properties [44], suggesting another clinical use for invasive oyster biomass.

The 24-methyl- and 24-ethylsterols, known as phytosterols (campesterol, brassicasterol, sitosterol, stigmasterol), as well as other sterols branched at C-24, all have well-established cholesterol-lowering properties [45,46], through the decrease in intestinal absorption of cholesterol. Phytosterols have also been shown to benefit cardiovascular and inflammatory conditions [47]. Since humans cannot synthesize phytosterols, they must be provided in the diet, and the high levels of these molecules in *C. gigas* throughout the year (more than 30%) makes them a potentially valuable source of these sterols.

## 3. Materials and Methods

### 3.1. Specimen Collection

Specimens of wild *Crassostrea gigas* were hand-collected at an oyster-farming site, La Couplasse, in Bourgneuf Bay (French Atlantic coast, 46–47° N, 1–2° W) in January, April, July and November 2007. They were immediately transported to the laboratory, where soft tissues were separated from the shells, pooled, homogenized with a blender (about 300 g tissue) and divided into three replicate lots. The homogenates were stored at −20 °C prior to analyses.

### 3.2. Chemicals

Standards of PLs (cardiolipin CL, phosphatidylcholine PC, phosphatidylethanolamine PE, phosphatidylinositol PI, phosphatidylglycerol PG, phosphatidylserine PS, lysophosphatidylcholine LPC, lysophosphatidylethanolamine LPE, sphingomyelin SPH) were sourced from Sigma-Aldrich (Saint-Quentin Fallavier, France). An authentic ceramide aminoethylphosphonate (CAEP) was kindly donated by Yanic Marty (CNRS, UBO, Brest, France).

### 3.3. Lipid Analyses

#### 3.3.1. Total Lipid Extraction and Separation of Lipid Classes

Lipid analyses were conducted as previously described [9]. Briefly, total lipids were extracted using dichloromethane/methanol (1:1, *v*/*v*) for 3 h at room temperature. After filtration on a Büchner funnel, the extract was washed with distilled water and evaporated to dryness and stored at −20 °C and under nitrogen prior to further analyses.

Lipid classes were separated on an open silica gel column (290 mm × 25 mm, 60 Å, 35–75 μm) with dichloromethane (neutral lipids), acetone (glycolipids) and methanol (PLs) as successive moving phases.

#### 3.3.2. High Performance Liquid Chromatography (HPLC) Analyses of Phospholipids

A modular UltiMate 3000 RS HPLC System (Thermo Scientific, Villebon sur Yvette, France) coupled with an evaporative light scattering detector (ELSD) Sedex 85 (Sedere S.A., Alfortville, France) was used to quantify the different PL classes using standard curves (10 μL of chloroform serially diluted solutions of CL, PE, PC, PG, SPH, LPC and LPE (1–10 μg), and CAEP, PI and PS (0.5–5 μg). For ELSD, nebulizer gas pressure (dried and filtrated air) was set to 3.5 bar and the detector was heated to 50 °C. The chromatographic separation was carried out using a Prevail™ silica column (150 mm × 4.6 mm, 3 μm particle diameter, Alltech Associates Inc., Lokeren, Belgium) heated at 25 °C with a 1.5 mL/min flow rate. PLs were eluted using gradient elution with two solvents: chloroform (A) and methanol/28% ammonia in water/chloroform (92:7:1, *v*/*v*/*v*) (B). Elution began at 0% B, increased to 20% in 3 min, then increased to 100% in 9 min and held for 3 min. The injection volume was 10 µL of a sample dilution in chloroform (2 mg/mL) and injections were performed in triplicates.

#### 3.3.3. Gas Chromatography-Mass Spectrometry (GC-MS)

All FAs of the total PLs were converted to the fatty acid methyl esters (FAMEs) by transmethylation with methanolic hydrogen chloride [9]. FAMEs were then converted to *N*-acyl pyrrolidides using pyrrolidine/acetic acid (5:1, *v*/*v*) in order to locate double bonds and branching [9]. Free sterols were isolated in the unsaponifiable fraction following saponification (2 M ethanolic potassium hydroxide) of neutral lipids. Sterols were converted to sterol acetates (SAs) by reaction with acetic anhydride and dried pyridine, for 12 h in darkness, at room temperature [48].

Separations of FAMEs, NAPs and SAs were achieved using a CP-Sil 5 CB low bleed MS capillary column (60 m × 0.25 mm I.D., 0.25 μm phase thickness—Chrompack, Middelburg, The Netherlands) under a constant flow rate (Helium-1 mL/min). For FAME analyses, the initial temperature of the GC-oven was 170 °C held for 4 min, with a subsequent increase (3 °C/min) to 300 °C. For NAP and SA analyses, the oven temperature was programmed at 200 °C (kept for 4 min—NAP), then increased to 310 °C (3 °C/min) and maintained at this temperature for 20 min for the NAPs and 25 min for the SAs.

### 3.4. Data Presentation

Data are presented as mean percentages for three replicates. The conventional indicator of dispersion about the mean is the standard deviation; we have followed this practice in the present work while awaiting the publication of recommendations for more appropriate presentation of this general type of data [49].

## 4. Conclusions

The high levels of high-quality lecithin observed in *C. gigas* (from 1 g (in spring) to 2.6 g (in winter)/100 g DW) make it a potential alternative source for commercial lecithin, a substance in high demand in the food industry [50]. This shows promise for nutritional and health applications, since a recent study has shown that marine PC rich in *n*-3 PUFAs has beneficial effects against colon cancer [51] and psoriasis [52,53,54,55]. The invasive oyster also contains lipids of interest, such as cardiolipin [56], plasmalogens and NMI FAs with effects against oxidative stress [38,39,57], *n*-3 PUFAS [58,59,60,61,62,63,64] and phytosterols with cholesterol-lowering effects, anti-inflammatory properties and as useful adjuvants in the reduction of cardiovascular risk [47] (Table 6).

This study is the second report showing the potential of an invasive mollusc as an alternative source of marine lecithin rich in PC and *n*-3 long-chain PUFAs [9]. Exploitation of this resource could constitute an avenue of remediation for these invasions.

## Figures and Tables

**Table 1:** Seasonal total tissue lipid content (TL) (% DW) and lipid class composition (% TL) of *Crassostrea gigas*. Values are the mean of three replicates (mean ± standard deviation).

Collection Season	Total Lipids (% DW)	Neutral Lipids (% TL)	Glycolipids (% TL)	Phospholipids (% TL)
Winter (January)	7.1 ± 0.5	40 ± 1	9 ± 2	50.4 ± 0.4
Spring (April)	8.6 ± 0.2	64.5 ± 0.7	7.4 ± 0.7	28.1 ± 0.9
Summer (July)	7.9 ± 0.1	39.3 ± 0.4	12.4 ± 0.4	48.0 ± 0.5
Autumn (November)	8.1 ± 0.4	50.3 ± 0.9	10.4 ± 0.8	39.3 ± 0.4

**Table 2:** Seasonal phospholipid class composition of *Crassostrea gigas* (% phospholipids). Values are the mean of three replicates (mean ± standard deviation).

Phospholipid Class	Winter	Spring	Summer	Autumn
Cardiolipin	6.8 ± 0.3	10.7 ± 0.3	7.2 ± 0.6	4.9 ± 0.5
Phosphatidylethanolamine	4.3 ± 0.2	18 ± 2	6.3 ± 0.3	5.3 ± 0.3
Ceramide aminoethylphosphonate	10 ± 1	24 ± 1	24.6 ± 0.4	22 ± 1
Phosphatidylserine	1.6 ± 0.9	4.0 ± 0.1	3.2 ± 0.4	5.7 ± 0.2
Phosphatidylcholine	74 ± 4	39.7 ± 0.4	53.2 ± 0.3	58.6 ± 0.6
Lysophosphatidylcholine	2.9 ± 0.3	3.8 ± 0.9	5.4 ± 0.5	3.3 ± 0.5

**Table 3:** Seasonal levels of phospholipid fatty acids of *Crassostrea gigas*. **^a^** ECLs (equivalent chain lengths) were determined using a CP-Sil 5 column CB. *i*, *iso*; *ai*, *anteiso*; br, branched.

Fatty Acids (Symbol)	ECL ^a^	Abundance (wt %)			
		Winter	Spring	Summer	Autumn
**Saturated Fatty Acids (SFAs)**					
14:0	14.00	1.05 ± 0.01	2.1 ± 0.1	1.46 ± 0.01	1.75 ± 0.04
4,8,12-Me_3_-13:0	14.49	0.3 ± 0.1	2.2 ± 0.1	1.73 ± 0.04	1.06 ± 0.04
15:0	15.00	1.2 ± 0.1	0.56 ± 0.09	0.61 ± 0.01	1.28 ± 0.01
*i*-16:0	15.60	0.5 ± 0.1	*	0.52 ± 0.02	0.44 ± 0.01
16:0	16.00	14.6 ± 0.5	16.8 ± 0.06	23.8 ± 0.1	19.1 ± 0.3
*i*-17:0	16.64	1.08 ± 0.01	0.5 ± 0.03	1.24 ± 0.03	0.84 ± 0.01
17:0	17.00	2.8 ± 0.4	1.41 ± 0.01	1.64 ± 0.03	2.4 ± 0.1
2-OH-16:0	17.18	1.6 ± 0.1	1.7 ± 0.1	0.9 ± 0.03	1.99 ± 0.07
*i*-18:0	17.64	0.36 ± 0.01	0.31 ± 0.01	0.88 ± 0.01	0.57 ± 0.02
18:0	18.00	5 ± 0.1	5.6 ± 0.2	7.32 ± 0.03	5.8 ± 0.2
**Total SFAs**		**28.5 ± 0.7**	**31.2 ± 0.4**	**40.1 ± 0.1**	**35.2 ± 0.7**
**Monounsaturated fatty acids (MUFAs)**					
9-16:1	15.74	1.75 ± 0.01	1.9 ± 0.1	1.18 ± 0.02	3.16 ± 0.01
7-Me-6(*Z*)-16:1	16.20	0.32 ± 0.01	n.d.	n.d.	0.20 ± 0.01
7-Me-6(*E*)-16:1	16.53	0.65 ± 0.01	0.22 ± 0.01	0.36 ± 0.03	0.42 ± 0.02
9-18:1	17.76	2.8 ± 0.1	0.94 ± 0.05	4.78 ± 0.04	2.41 ± 0.07
11-18:1	17.81	4.5 ± 0.1	5.8 ± 0.2	2.46 ± 0.03	5.72 ± 0.06
3-19:1	18.58	1.6 ± 0.1	1.53 ± 0.05	1.36 ± 0.01	2.5 ± 0.01
11-20:1	19.68	1.6 ± 0.1	3.34 ± 0.07	7.19 ± 0.03	2.94 ± 0.03
13-20:1	19.73	5.8 ± 0.2	5.43 ± 0.05	2.16 ± 0.01	5.8 ± 0.1
**Total MUFAs**		**19.0 ± 0.5**	**19.2 ± 0.6**	**19.5 ± 0.05**	**23.2 ± 0.3**
**Polyunsaturated fatty acids (PUFAs)**					
18:4*n*-3	17.54	1.01 ± 0.01	0.9 ± 0.02	1.00 ± 0.01	0.9 ± 0.01
18:2*n*-6	17.66	0.3 ± 0.1	0.84 ± 0.04	2.84 ± 0.05	0.67 ± 0.01
20:4*n*-6	19.24	4.2 ± 0.1	2.07 ± 0.01	3.35 ± 0.04	3.67 ± 0.01
20:5*n*-3	19.34	9.3 ± 0.1	14.5 ± 0.1	7.53 ± 0.03	9.54 ± 0.01
20:3*n*-7	19.49	0.5 ± 0.01	0.73 ± 0.03	0.40 ± 0.01	0.45 ± 0.01
20:2*n*-9,12	19.52	0.9 ± 0.1	1.28 ± 0.04	1.10 ± 0.01	1.06 ± 0.05
22:6*n*-3	21.12	7.8 ± 0.2	5.51 ± 0.09	9.50 ± 0.03	7.72 ± 0.09
22:4*n*-6	21.19	1.42 ±0.01	1.4 ± 0.05	2.36 ± 0.01	1.17 ± 0.06
22:5*n*-3	21.28	0.38 ± 0.01	0.30 ± 0.01	0.45 ± 0.01	0.37 ± 0.01
22:2*n*-9,15	21.40	1.31 ± 0.01	0.99 ± 0.01	1.98 ± 0.02	1.1 ± 0.01
22:2*n*-7,15	21.46	9.6 ± 0.3	9.02 ± 0.13	4.74 ± 0.05	7.4 ± 0.2
**Total PUFAs**		**36.7 ± 0.7**	**37.5 ± 0.5**	**35.2 ± 0.3**	**34.1 ± 0.6**
**Fatty aldehyde dimethylacetals (DMAs)**					
16:0	16.48	0.68 ± 0.01	0.56 ± 0.08	0.25 ± 0.01	0.39 ± 0.02
br-17:0	17.12	0.8 ± 0.1	0.39 ± 0.04	0.24 ± 0.01	0.28 ± 0.04
br-17:0	17.22	0.25 ± 0.01	*	0.27 ± 0.01	0.2 ± 0.01
17:0	17.48	0.83 ± 0.01	0.59 ± 0.08	*	0.49 ± 0.07
br-18:0	18.10	0.9 ± 0.1	n.d.	n.d.	n.d.
br-18:0	18.22	0.82 ± 0.01	0.39 ± 0.02	n.d.	0.38 ± 0.03
18:0	18.48	6.6 ± 0.2	6 ± 2	1.49 ± 0.02	2.97 ± 0.03
br-19:0	19.22	0.33 ± 0.01	0.19 ± 0.01	*	0.26 ± 0.02
br-20:1	20.06	1 ± 0.1	0.42 ± 0.06	*	0.28 ± 0.06
br-20:1	20.10	0.41 ± 0.01	0.37 ± 0.08	0.49 ± 0.01	0.59 ± 0.09
20:0	20.17	1.9 ± 0.2	1.66 ± 0.04	0.65 ± 0.02	0.63 ± 0.03
**Total DMAs**		**14.5 ± 0.4**	**10.6 ± 0.2**	**3.39 ± 0.05**	**6.5 ± 0.3**

Minor FAs as traces (<0.2%) (ECL): *i*-15:0 (14.62); 4-16:1 (15.70); *ai*-17:0 (16.73); *i*-19:0 (18.61); *ai*-19:0 (18.72); br-20:0 (19.38); 21:2*n*-8,14 (19.91); 22:0 (22.00); 5-24:1 (23.27); 24:0 (24.00). Values are the means of three replicates (mean ± standard deviation. n.d., not detected, and * <0.2%).

**Table 4:** Comparison between the present study and the literature for the major PUFAs.

Species	EPA % TL FA	DHA % TL FA	NMI % TL FA	Country	References
*C. gigas*	10.8–15.2	10.3–15.5	-	Ireland	[21]
*C. gigas*	16.4–25.5	15.6–21.3	3.9–7.7	Germany	[20]
*C. rhizophorae*	17.9–19.7	19.7–35.8	-	Brazil	[33]
**Species**	**% PL FA**	**% PL FA**	**% PL FA**	**Country**	**References**
*C. gigas*	7.5–15.4	5.5–9.5	6.8–10.9	France	Present study
*C. gigas*	13.8–22.4	9.9–17.6	3.3–7.3	Spain	[22]
*C. gigas*	12.3 in muscle	17.3 in muscle	4.1 in muscle	France	[24]
*C. gigas*	18.6–21.7 in PC	13.1–15.8 in PC	2.7–3.5 in PC	France	[23]
*C. corteziensis*	10.3–17.4	22.5–32.5	7.8–12.8	Mexico	[34]
*O. edulis*	7.6–17.4	8.2–18.5	2.8–13	Spain	[37]

**Table 5:** Seasonal composition of the total free sterols of *Crassostrea gigas*. X1, X2: unidentified sterols; Values are the mean of three replicates (mean ± s.d.); s.d., standard deviation; n.d., non detected. % phytosterols calculated with respect to total sterols.

Systematic Names	Trivial Names	% Total Lipids			
		Winter	Spring	Summer	Autumn
24-*nor*-Cholesta-5,22*E*-dien-3β-ol	24-*nor*-Dehydrocholesterol	5.2 ± 0.1	5.65 ± 0.1	3.57 ± 0.05	4.9 ± 0.2
24-*nor*-5α-Cholest-22*E*-en-3β-ol	24-*nor*-Dehydrocholestanol	0.51 ± 0.06	n.d.	0.47 ± 0.02	0.7 ± 0.07
Cholesta-5,22*Z*-dien-3β-ol	22*Z*-Dehydrocholesterol	1.7 ± 0.3	1.92 ± 0.08	0.9 ± 0.1	1.7 ± 0.2
Cholesta-5,22*E*-dien-3β-ol	22*E*-Dehydrocholesterol	8.94 ± 0.07	6.3 ± 0.2	5.6 ± 0.1	7.5 ± 0.4
5α-Cholest-22*E*-en-3β-ol	22-Dehydrocholestanol	1.2 ± 0.2	n.d.	0.8 ± 0.2	1.4 ± 0.2
Cholest-5-en-3β-ol	Cholesterol	32.1 ± 0.7	35.8 ± 0.4	21.4 ± 0.3	30.3 ± 0.4
5α-Cholestan-3β-ol	Cholestanol	3.94 ± 0.08	6.7 ± 0.7	4 ± 0.1	4.7 ± 0.2
24-Methylcholesta-5,22*E*-dien-3β-ol	Brassicasterol/Crinosterol	12.8 ± 0.7	12.6 ± 0.3	9.19 ± 0.05	10.7 ± 0.2
4α-Methyl-5α-cholest-7-en-3β-ol	Lophenol	0.91 ± 0.08	n.d.	n.d.	n.d.
X1 (Δ° C28:0)	--	0.5 ± 0.2	n.d.	1.71 ± 0.05	n.d.
24-Methylcholesta-5,24(28)-dien-3β-ol	24-Methylenecholesterol	14 ± 0.2	12.9 ± 0.5	8.35 ± 0.03	10.7 ± 0.1
24-Methylcholest-5-en-3β-ol	Campesterol/22,23-Dihydrobrassicasterol	6.24 ± 0.07	6.93 ± 0.03	6 ± 0.1	5.5 ± 0.1
5α-24-Ethylcholest-25-en-3β-ol	5α-Poriferast-25-en-3β-ol/25-Dehydroporiferastanol	0.53 ± 0.07	n.d.	n.d.	n.d.
5α-24-Ethylcholesta-22,24(25)-dien-3β-ol	5α-Porifera-22,24(25)-dien-3β-ol	1.2 ± 0.1	n.d.	1.11 ± 0.01	1.1 ± 0.2
24-Ethylcholest-5,22*E*-dien-3β-ol	Poriferasterol/Stigmasterol	1.78 ± 0.03	1.7 ± 0.2	1.67 ± 0.04	1.7 ± 0.1
4,24-Dimethylcholesta-5,7,24(28)-trien-3β-ol	4-Methyl-5α-Ergosta-24(28)-en-3β-ol	0.4 ± 0.03	n.d.	7.8 ± 0.1	5.6 ± 0.3
24-Ethylcholest-5-en-3β-ol	β-Sitosterol/Clionasterol	5.4 ±0.3	9.9 ± 0.5	10.2 ± 0.2	6.0 ± 0.4
24-Ethyl-5α-cholest-22*E*-en-3β-ol	Poriferastanol/Stigmastanol	2.8 ± 0.2	2.6 ± 0.2	1.6 ± 0.2	3.5 ± 0.2
24-Ethylcholesta-5,24(28)-dien-3β-ol	Fucosterol	n.d.	n.d.	1.23 ± 0.04	0.65 ± 0.04
4-Methyl-24-ethyl-5α-cholesta-7-en-3β-ol	4-Methyl-5α-Porifera-7-en-3β-ol/24-Ethyllophenol	n.d.	n.d.	2.90 ± 0.05	0.96 ± 0.08
X2 (Δ° C30:0)	--	n.d.	n.d.	0.95 ± 0.04	n.d.
4-Methyl-24-ethyl-cholesta-5-en-3β-ol	4-Methyl-Porifera-5-en-3β-ol	n.d.	n.d.	0.73 ± 0.09	0.61 ± 0.09
% Phytosterols		30.8	33.7	33.4	30.1

**Table 6:** Potential economic, nutritional, and health benefits of *C. gigas* lipids.

Lipids	Potential Benefits	References
Lecithin (with associated PUFAs)	Protection against colon cancer	[51,54,55]
	Treatment of psoriasis	[52]
	High-quality source for food industry	[49]
CAEP	Implication in some haemocyte functions	[28,30]
Cardiolipin	Optimization of mitochondrial respiratory	[56]
	performance	[25,26]
*n*-3 Polyunsaturated FAs (EPA, DHA)	Improve efficacy and tolerability of cancer chemotherapy	[59]
	Cancer prevention	[58]
	Neuroprotective efficacy	[60]
	Cardiovascular disease protection	[61]
	Improvement of some obesity-associated metabolic syndrome features (Type 2 diabetes)	[62,63]
	Anti-inflammatory effect	[64]
Diunsaturated NMI FAs	Resistance to oxidative stress and microbial lipases	[38,57]
Plasmalogens	Countering oxidative stress	[38,39]
Phytosterols	Cholesterol-lowering action	[45,46]
	Reduction in cardiovascular risk	[47]
	Anti-inflammatory effect	[45]

## Abbreviations

CAEP	ceramide aminoethylphosphonate
DHA	*n*-3 docosahexaenoic acid
DMA(s)	dimethylacetal(s)
DW	dry weight
EPA	*n*-3 eicosapentaenoic acid
ELSD	evaporative light scattering
FA(s)	fatty acid(s)
FAME(s)	fatty acid methyl ester(s)
GC-MS	gas chromatography-mass spectrometry
HPLC	high performance liquid chromatography
LPC	lysophosphatidylcholine
LPE	lysophosphatidylethanolamine
MUFA(s)	monounsaturated fatty acid(s)
NAP(s)	*N*-acyl pyrrolidide(s)
NMID	non-methylene-interrupted dienoic
PC	phosphatidylcholine
PE	phosphatidylethanolamine
PG	phosphatidylglycerol
PI	phosphatidylinositol
PS	phosphatidylserine
PUFA(s)	polyunsaturated fatty acid(s)
PL(s)	phospholipid(s)
SA(s)	sterol acetate(s)
SFA(s)	saturated fatty acid(s)
SPH	sphingomyelin
TL	total lipids
